# The Emerging Role of Heat Shock Factor 1 (HSF1) and Heat Shock Proteins (HSPs) in Ferroptosis

**DOI:** 10.3390/pathophysiology30010007

**Published:** 2023-03-14

**Authors:** Iman Aolymat, Ma’mon M. Hatmal, Amin N. Olaimat

**Affiliations:** 1 Department of Anatomy, Physiology and Biochemistry, Faculty of Medicine, The Hashemite University, P.O. Box 330127, Zarqa 13133, Jordan; 2 Department of Medical Laboratory Sciences, Faculty of Applied Medical Sciences, The Hashemite University, P.O. Box 330127, Zarqa 13133, Jordan; 3 Department of Clinical Nutrition and Dietetics, Faculty of Applied Medical Sciences, The Hashemite University, P.O. Box 330127, Zarqa 13133, Jordan

**Keywords:** ferroptosis, Xc-GSH-GPX4 axis, heat shock proteins, HSPs, HSF1, molecular chaperones, stress response

## Abstract

Cells employ a well-preserved physiological stress response mechanism, termed the heat shock response, to activate a certain type of molecular chaperone called heat shock proteins (HSPs). HSPs are activated by transcriptional activators of heat shock genes known as heat shock factors (HSFs). These molecular chaperones are categorized as the HSP70 superfamily, which includes HSPA (HSP70) and HSPH (HSP110) families; the DNAJ (HSP40) family; the HSPB family (small heat shock proteins (sHSPs)); chaperonins and chaperonin-like proteins; and other heat-inducible protein families. HSPs play a critical role in sustaining proteostasis and protecting cells against stressful stimuli. HSPs participate in folding newly synthesized proteins, holding folded proteins in their native conformation, preventing protein misfolding and accumulation, and degrading denatured proteins. Ferroptosis is a recently identified type of oxidative iron-dependent cell demise. It was coined recently in 2012 by Stockwell Lab members, who described a special kind of cell death induced by erastin or RSL3. Ferroptosis is characterized by alterations in oxidative status resulting from iron accumulation, increased oxidative stress, and lipid peroxidation, which are mediated by enzymatic and non-enzymatic pathways. The process of ferroptotic cell death is regulated at multiple, and it is involved in several pathophysiological conditions. Much research has emerged in recent years demonstrating the involvement of HSPs and their regulator heat shock factor 1 (HSF1) in ferroptosis regulation. Understanding the machinery controlling HSF1 and HSPs in ferroptosis can be employed in developing therapeutic interventions for ferroptosis occurrence in a number of pathological conditions. Therefore, this review comprehensively summarized the basic characteristics of ferroptosis and the regulatory functions of HSF1 and HSPs in ferroptosis.

## 1. Introduction

In order to maintain homeostasis, biological cells employ a well-preserved stress response mechanism termed a heat shock response. This mechanism entails the activation of molecular chaperones, known as heat shock proteins (HSPs) by transcriptional activators of heat shock genes, known as heat shock factors (HSFs) [1]. HSPs assemble into oligomeric complexes and interact with cellular proteins to maintain proteostasis [2,3]. As molecular chaperones, HSPs sustain the functional conformation of their client proteins by rescuing misfolded proteins and restoring their native conformation, holding proteins in soluble state and preventing their precipitation, or directing them for proteolysis [4].

Cell death is an important feature of living organisms to preserve homeostasis and get rid of damaged cellular structures. Ferroptosis is a recently identified type of oxidative iron-dependent cell demise. Ferroptosis differs from other recognized forms of cell death, such as apoptosis and necrosis, in terms of morphology and biochemical properties [5]. Reactive oxygen species (ROS) production and severe membrane lipid peroxidation that are iron-dependent are hallmarks of ferroptotic cell death [5]. This form of cellular death has been described in several pathophysiologies of various disorders such as neurodegenerative disorders, cancers, and kidney diseases [6].

## 2. Heat Shock Proteins (HSPs) and Their Transcriptional Regulator Heat Shock Factor 1 (HSF1)

In order to maintain homeostasis, biological cells adopt an adequately established stress response mechanism, termed the heat shock response. This mechanism involves the induction of molecular chaperones, known as HSPs, via transcriptional activators of heat shock genes called HSFs, including HSF1, HSF2, HSF3, HSF4, HSFX, and HSFY [1]. This highly conserved response is important to prevent the formation of cytotoxic denatured and insoluble precipitated proteins. Among the HSFs, HSF1 plays a major role in the heat shock response. On activation, HSF1 interacts with heat shock elements within the target heat shock gene promotors, inducing various HSPs expression. The activated HSPs either support the refolding of misfolded proteins or target them for proteasomal degradation [7].

**Figure 1:**
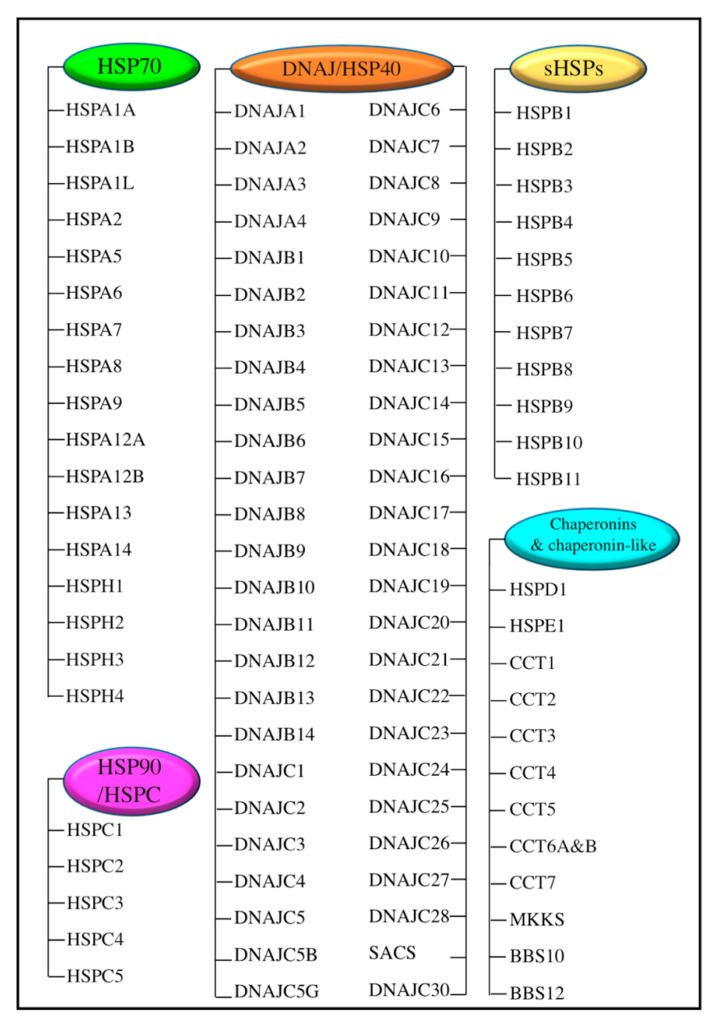
Kampinga et al., 2009 classifications for the heat shock protein families.

HSP families are composed of peptides that range in size from 10 to more than 100 kDa and are found in different cellular compartments. According to the HUGO Gene Nomenclature Committee, Kampinga et al. [8] classified HSPs into the HSP70 superfamily (including HSPA (HSP70) and HSPH (HSP110) families); the DNAJ (HSP40) family; the HSPB family (small heat shock proteins (sHSPs)); chaperonins and chaperonin-like proteins; and other heat-inducible protein families (Figure 1) [8]. Major HSPs that have a large molecular weight, such as HSP90 and HSP70, have ATPase activity that is regulated by other accessory non-client proteins, called co-chaperones. Co-chaperones play a fundamental role in inducing HSP proteins’ ATPase activity. The hydrolysis of ATP catalyzed by co-chaperones resulted in HSPs’ conformational changes and increased their affinity to their target proteins, thus improving their protein-folding function [9,10]. The sHSPs family is composed of low molecular weight proteins (12–43 kDa) with a highly conserved central domain, containing 80–100 amino acids that comprise the α-crystalline domain [11]. A distinctive feature of sHSPs is the assembly of homo- or hetero-oligomeric complexes that aid these proteins in serving as molecular chaperones [12]. The αA crystal protein (CRYAA, also known as heat shock protein beta 4 (HSPB4)), the αB crystal protein (CRYAB, also known as heat shock protein beta 5 (HSPB5)), the heat shock protein beta 1 (HSPB1, also known heat shock protein 27 (HSP27), and the heat shock protein 25 (HSP25) are the most studied in the sHSPs family. HSPs play a fundamental role in proteostasis by interacting with cellular proteins to maintain their target proteins in a folding-competent state; to rescue misfolded proteins and restore their native conformation; to prevent peptide precipitation; or to direct the proteins for proteolysis [4].

In the past ten years, there has been a revolution in the understanding of ferroptosis’ physiology and pathophysiology. It was reported that the heat shock response and HSP family members are involved in ferroptosis due to the stressful nature of iron-dependent oxidative damage to membrane lipids. More research has emerged in recent years demonstrating the involvement of HSF1 and HSPs in the ferroptosis pathophysiology, where they participated in distinct ways in ferroptosis regulation. However, the available knowledge on the function of HSF1 and HSPs in ferroptotic cell death is still insufficient. Understanding the role of HSF1 and HSPs in ferroptotic cell death can be employed by developing therapeutic interventions for ferroptosis occurrence in a number of pathological conditions, particularly cancer. This work comprehensively reviewed the basic features of ferroptosis and the regulatory functions of HSF1 and HSPs in ferroptosis in order to offer new insights into the potential roles and mechanisms of HSF1 and HSPs in controlling ferroptosis.

## 3. Ferroptosis

### 3.1. Overview

Cell death is an active process to maintain homeostasis and eradicate damaged cellular structures. Based on the morphology, cell death was initially categorized as type-I cell death (apoptosis), type-II cell death (autophagy), and type-III cell death (necrosis) [13]. The more recent characterization by the Cell Death Nomenclature Committee has suggested that cell death is predominantly categorized as two main types, regulated cell death (RCD) and accidental cell death (ACD) [13]. The RCD is a controlled form of cell death that maintains the homeostasis mediated by signaling pathways in order to trigger intrinsic cell death mechanisms that occur during physiological cell turnover and adaptation to stressful conditions. The RCD can be attenuated by therapeutic and genetic interventions. The RCD includes the apoptosis, ferroptosis, pyroptosis, necroptosis, parthanatos, lysosome-dependent cell death, autophagy-dependent cellular demise, entotic cellular demise, NETotic cellular demise, and immunogenic cellular demise subtypes [13]. In contrast, ACD is an uncontrolled cellular death that occurs due to harmful external stimuli [13].

Ferroptosis is a newly recognized oxidative iron-dependent kind of RCD. Ferroptosis varies from other modes of programmed cell death, such as apoptosis and necrosis, in terms of its morphology and biochemical characteristics [14]. Morphologically, ferroptosis is associated with a change in mitochondrial size and the hyperdensity of the mitochondrial membrane. Biochemically, ferroptosis is characterized by alterations in oxidative status resulting from iron accumulation, increased oxidative stress, and lipid peroxidation [15]. This kind of RCD was identified in different vital organs, such as the brain, kidneys, and heart [14]. Despite its essential role in cellular survival and maintaining homeostasis in physiological conditions, ferroptosis was identified in several pathologic conditions as well, such as neuropathies, kidney injury, diabetes mellitus, ischemia-reperfusion injury, and cancer [16]. 

### 3.2. Basic Features of Ferroptosis

Biochemically, the accumulation of free iron and severe lipid peroxidation that result in cell membrane injury are the key features of ferroptotic cell death. Iron accumulation is associated with a reduced efficiency in the antioxidant system and thus an increased production of ROS via the Fenton reaction [17], initiating oxidative damage to different cellular structures. Alternatively, high iron levels stimulate iron-containing enzymes, such as arachidonate lipoxygenase (ALOX) and prolyl hydroxylase (PHD), mediating excessive lipid peroxidation [5]. Therapeutic interventions, such as iron chelators and gene-targeted therapy that reduce iron overload, have been recognized as measures that inhibit ferroptotic cell death [6]. Lipid peroxidation is a form of oxidative breakdown reaction caused by high iron levels, and it affects predominantly polyunsaturated-fatty-acid (PUFA) constituents of plasma membranes. During this reaction, lipid hydroperoxides and reactive aldehydes are produced, which mediate the destruction of the cell membrane [18]. ALOX is one of the crucial enzymes that is important for the oxygenation of PUFAs, promoting lipid peroxidation and ferroptosis [19]. Moreover, some mitochondrial membrane electron-transfer proteins, such as nicotinamide adenine dinucleotide phosphate (NADPH) oxidase, play a major role in lipid peroxidation via ROS synthesis, executing ferroptotic cell death [20].

Ferroptotic cell demise is predominantly characterized by a significant structural dysregulation of the mitochondria, the organelle responsible for energy production and ROS generation. Examples of mitochondrial morphological changes that occur during ferroptosis are mitochondrial shrinkage; reduced or missing mitochondrial cristae; mitochondrial condensation; mitochondrial membrane hyperdensity; and mitochondrial membrane rupture [6,13]. However, researchers have observed morphological changes in ferroptotic cell death that overlap with the morphological features of other types of cell death, such as a break in plasma membrane barrier integrity, a swelling of the cytoplasm, chromatin condensation, remarkable autophagosome synthesis, and cell rounding and detachment [6].

Multiple genes and proteins were identified as biomarkers for ferroptosis. For example, the induction of ferroptosis by erastin (a small molecule implicated in cancer therapy) has been associated with an increased expression of prostaglandin-endoperoxide synthase, which is also known as cyclooxygenase-2 (PTGS2/COX2) [21]. This enzyme catalyzes the production of prostaglandins from arachidonic acid. Another enzyme employed as a biomarker for ferroptosis is the Acyl-Coenzyme A (CoA) synthetase long-chain family member 4 (ACSL4) [22], which is involved in the esterification of CoA to free acids. ACSL4 contributes to ferroptosis by generating PUFAs, which are susceptible to lipid peroxidation, and it has been demonstrated that pharmacological suppression of this enzyme was a powerful inhibitor of ferroptotic cell death [23]. Moreover, some studies have shown that ACSL4 was not a mandatory driver for ferroptosis, and that p53 was another regulator for ferroptosis when ACSL4-null cells were resistant to erastin-mediated ferroptosis but susceptible to p53-driven ferroptosis [24]. Since increased levels of lipid peroxides favor ferroptotic cell death, the induction of free-radical scavenging systems, such as the glutathione (GSH) [5] and coenzyme Q10 (CoQ10) systems [25], has been found to suppress ferroptotic cell death.

Ferroptosis is associated with several inflammatory and immunological reactions that cause cell death by diverse signaling pathways. Lipid-peroxidation-mediated ferroptosis caused the death of certain immune cells, such as T-cells, with the consequent loss of immune tolerance [26]. Moreover, cells suffering from ferroptosis released either damage-associated molecular-patterned (DAMP) molecules, such as the high-mobility group box 1 (HMGB1) and DNA, or a variety of lipid peroxidation products, such as 4-hydroxynonenal, (4-HNE), oxidized phospholipids (oxPLs), prostaglandin E2 (PGE2), and leukotrienes, which then triggered various inflammatory reactions [6]. 

### 3.3. Regulation Mechanism Ferroptosis

#### 3.3.1. Iron Metabolism

Iron is a major element that plays a crucial role in numerous mammalian functions. Physiologically, iron functions as a cofactor for several proteins and enzymes that are involved in oxygen transport (such as hemoglobin), antioxidant mechanisms (such as catalases and peroxidases), and the electron transport chain of mitochondria (such as cytochromes). It is also vital for DNA synthesis, cell cycling, and the biosynthesis of steroid hormones [27]. Iron is present in two oxidation states, the divalent ferrous ion (Fe^2+^) and the trivalent ferric ion (Fe^3+^), which enables it to play a major role in electron-transport systems [27]. 

**Figure 2:**
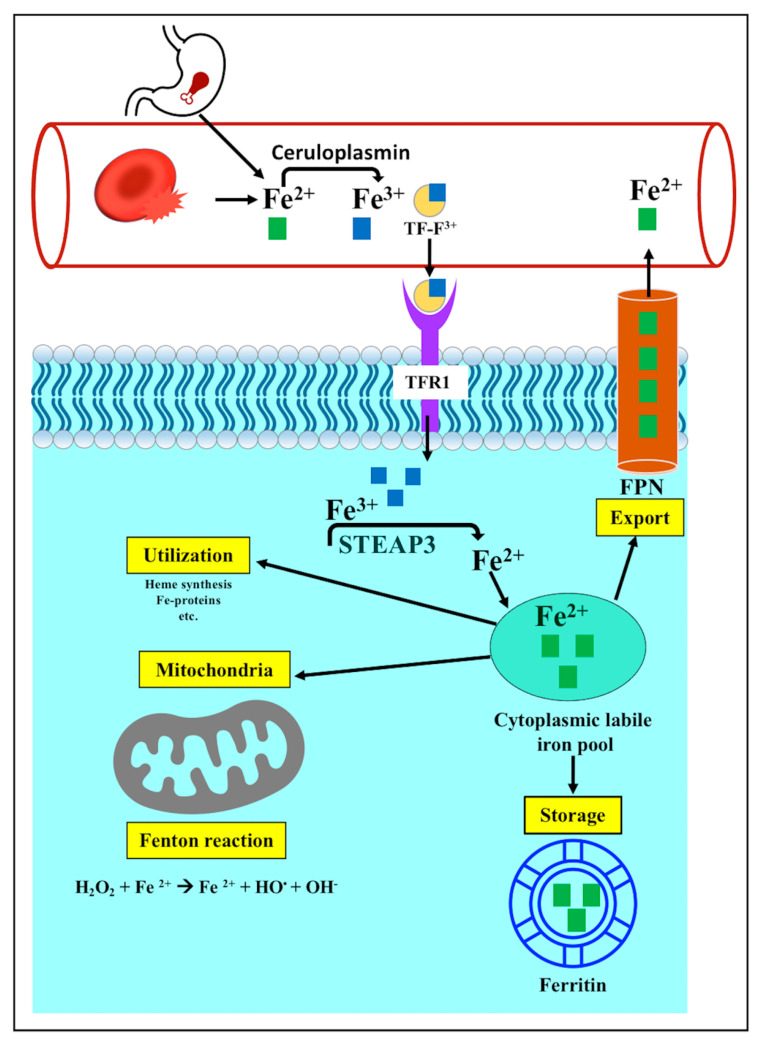
Iron metabolism in ferroptosis. Iron homeostasis depends on the balance between iron uptake, utilization, storage, and export to the circulation. Iron in the blood is either absorbed through the intestines or released through the breakdown of red blood cells. It is present in the Fe^2+^ oxidation state. Ceruloplasmin converts Fe^2+^ to Fe^3+^, which can bind with transferrin (TF). Through TF receptor 1 (TFR1), the TF-bound ferric ion travels to the cell. Fe^3+^ is converted into Fe^2+^ inside the cell by the enzyme six-transmembrane epithelial antigen of the prostate 3 (STEAP3), which is then released into the cytoplasmic labile iron pool. From this pool, iron is either utilized in various biological functions, supplied to the mitochondria, stored in ferritin, or exported via ferroportin1 (FPN) to the circulation.

Iron in the blood is the result of either intestinal absorption or the destruction of red blood cells, and it is present in the Fe^2+^ oxidation state. Fe^2+^ is oxidized via ceruloplasmin to Fe^3+^, which can bind transferrin (TF). The TF-bound ferric ion moves to the cells via TF receptor 1 (TFR1). Inside a cell, the enzyme’s six-transmembrane epithelial antigen of the prostate 3 (STEAP3) converts Fe^3+^ into Fe^2+^, which is released into a cytoplasmic labile iron pool. From this pool, iron is either stored as ferritin heavy chain 1 (FTH1) and ferritin light chain (FTL), which is not toxic to the cell; provided to the mitochondria; or alternatively, exported via ferroportin1 (FPN) to the blood circulation [16] (Figure 2). 

Iron hemostasis in mammals is maintained by different mechanisms, involving iron regulatory proteins (IRP1 and IRP2) and iron-responsive element (IRE) signaling pathway. Depending on the level of iron within the cell, IRP1 and IRP2 regulate the expression of the iron metabolism genes by binding to the RNA stem-loops containing an IRE in the untranslated region. This binding controls the translation and stability of the target mRNA. TF, and TFR1, and the ferritin are targets for IRP1 and IRP2 [28].

Many pathological disorders develop from abnormalities in iron metabolism, resulting in iron accumulation or iron deficiency. Iron surfeit caused by either increased iron absorption or reduced iron consumption has been associated with iron accumulation and the induction of oxidative destruction of the membrane lipids, which is a characteristic feature of ferroptotic cell death [29]. Several previous observations have linked the increased iron accumulation to ferroptotic cell demise. For example, reducing intracellular iron accumulation via the inactivation of TFR1 can limit erastin-dependent ferroptosis [30]. Sun et al. found that hepatocellular carcinoma cells with genetically depleted-FTH1 exhibited erastin-mediated ferroptotic cell death [31]. Kown et al. reported that cancer cells did not undergo erastin-mediated ferroptosis when heme oxygenase (HO), an enzyme that produces intracellular iron from heme breakdown, was inhibited [32]. Furthermore, the genetic modulation of the iron transporter FPN and the solute carrier family 40 member 1 (SLC40A1) that exports iron extracellularly has also added to the available knowledge about the regulatory function of iron homeostasis in the development of ferroptosis. Ma et al. reported that the increased expression of FPN mitigated ferroptosis in breast cancer cells, while a reduced FPN expression increased ferroptotic cell death [33]. Additionally, it has been shown that the suppression of nuclear translocation of metal regulatory transcription factor 1 (MTF1), which normally mediates the expression of FPN and TF and reduces the level of free iron within the cell, has been associated with an increased risk of ferroptotic cell death [34]. In addition, iron chelators were found to have a protective effect against ferroptotic cell death, providing additional proof of the involvement of iron in ferroptosis [35]. More recent studies have suggested a link between autophagy and ferroptotic cell death. The autophagic destruction of FTH1 and FTL, which is called ferritinophagy, increases the level of unbound iron in cancer cells, making cells more susceptible to ferroptosis [36]. Moreover, the suppression of autophagy caused by the genetic modulation of autophagy-related genes has been associated with inhibition of ferroptotic cell death, and vice versa [37,38]. Therefore, these observations have indicated that iron balance is a major regulator of ferroptotic cell death, and any disruption of iron absorption, transport, consumption, or storage will lead to abnormalities in systemic iron homeostasis. It has been revealed that excess iron has a cytotoxic effect by increasing the formation of oxygen radicals via the mitochondrial Fenton reaction (Figure 2), which mediates the oxidative stress destruction of the cell membrane through lipid peroxidation (Figure 2) [39]. 

#### 3.3.2. Lipid Metabolism

Lipids are the principal components of the cell membrane, and abnormal lipid metabolism is tightly tied to ferroptosis. Cells absorb the necessary fatty acids from the blood circulation to synthesize various types of lipids. In ferroptosis, the process of the iron-dependent oxidation of lipids, which is also known as lipid peroxidation, is associated with multiple free radical reactions [40]. PUFAs, such as arachidonic acid and adrenic acid, which are enzymatically incorporated into membrane phospholipids and control its structure and fluidity, are the major targets for lipid peroxidation reactions [40].

**Figure 3:**
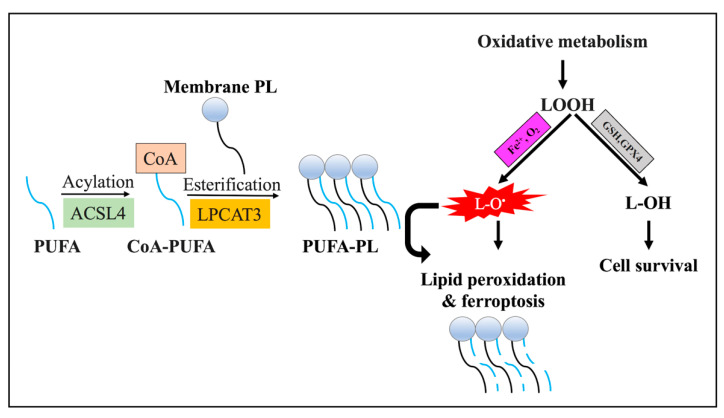
Mechanism of ferroptosis. ACSL4 and LPCAT3 enzymes are required for the initiation of ferroptosis. ACSL4 catalyzes the acylation of PUFAs, which are then inserted into membrane PLs via the action of LPCAT enzymes. PUFA-containing membrane PLs are affected by peroxidation that leads to the destruction of the plasma membrane and ferroptosis. Abbreviations: ACSL4, acyl-Coenzyme A (CoA) synthetase long-chain family member 4; GPX4, glutathione peroxidase 4; GSH, glutathione; L-O•, lipid radicals; L-OH, lipid alcohols; LOOH, lipid hydroperoxides; LPCAT3, lysophosphatidylcholine acyltransferase 3; PL, phospholipid; PUFAs, polyunsaturated fatty acids.

There are two major enzymes, the acyl-CoA synthetase long-chain family member 4 (ACSL4) and lysophosphatidylcholine acyltransferase (LPCAT), and these are required for the initiation of ferroptosis. These two enzymes are involved in the generation of PUFA-containing phospholipid membranes, which are affected by peroxidation [18] (Figure 3). According to recent research, ferroptosis is modulated by changes in the expression levels of ACSL4 and LPCAT. It has been identified that the execution of ferroptotic cell death is driven by the induction of ACSL4, which is considered a biomarker for ferroptosis [22,41]. Conversely, the suppression of ACSL4 or LPCAT activities interferes with ferroptotic cell death [22,23,40,42]. Accordingly, targeting ACSL4 or LPCAT to modulate ferroptosis in tumor therapy could affect tumor development and survival. Collectively, ACSL4 and LPCAT could be exploited as prognostic biomarkers and potential therapeutic targets. 

In accordance with the unique role of PUFAs in ferroptotic cell death, more recent studies have shown that the dietary supplementation of PUFAs was sufficient to trigger ferroptotic cell death in different models [43]. However, exogenous monounsaturated-fatty-acid (MUFA) treatment showed a protective effect against ferroptosis [44,45]. Through the action of the ACSL3 enzyme, MUFAs are incorporated into the phospholipid bilayer of the membrane, reducing the level of the PUFAs prone to lipid peroxidation. Additionally, it has been found that MUFA administration lowered the level of lipid ROS accumulation at the membrane [44].

The lipid peroxidation process is tightly controlled by two types of reactions: enzymatic and non-enzymatic. Both reactions affect the membrane-associated PUFAs and interfere with cellular homeostasis through the disruption of the biological membrane or the formation of protein adduction [46]. The enzymatic peroxidation reactions of lipids are controlled by the iron-containing ALOX enzymes family members, which include ALOXE3, ALOX5, ALOX12, ALOX12B, ALOX15, and ALOX15B enzymes [47]. The enzymatic reaction triggers ferroptotic cell demise via the generation of the membrane-damaging PUFA hydroperoxides (Figure 3) and further reactive aldehydes species (such as 4-HNE) or malondialdehyde (MDAs) [48]. Studies have found that the suppression of the ALOX enzymes was associated with enhanced cellular resistance to ferroptosis [24,49]. Alternatively, more recently identified oxidoreductases, such as NADPH-cytochrome P450 reductase (POR) and NADH-cytochrome b5 reductase (CYB5R1), are involved in enzymatic lipid peroxidation [50].

In contrast, the non-enzymatic lipid peroxidation mechanism involves an iron-catalyzed multi-stage oxidation process that produces free radicals, resulting in the oxidation of membrane-associated PUFAs [51]. The process initially generates hydroxyl and peroxyl radicals via the Fenton reaction (Figure 2), which further triggers the oxidation of susceptible PUFA-containing membranes and produces lipid-peroxyl radicals, lipid peroxides, and other radicals (Figure 3) [51]. Therefore, the use of antioxidants and iron chelators, such as desferrioxamine (DFO), which are able to reduce ROS production and the liable iron, respectively, has been shown to minimize the peroxidation of lipids [52]. 

To date, the mechanism of lipid-peroxidation-mediated ferroptotic cell death is not well understood, and several proposed mechanisms have been established. Firstly, ROS associated with lipid peroxidation causes the destruction of different biological targets, such as DNA, amino acids, and peptides [53]. Secondly, the reactive aldehydes species, such as 4-HNE and MDA, can interfere with the physiological function of various cellular proteins throughs crosslinking [54]. Lastly, the dysregulation of biological membranes due to the peroxidation of its components has been associated with structural changes, such as the formation of membrane pores and increased membrane permeability [55,56].

#### 3.3.3. Disruption of Endogenous Redox Homeostasis System, the Xc-GSH-GPX4 Axis

**Figure 4:**
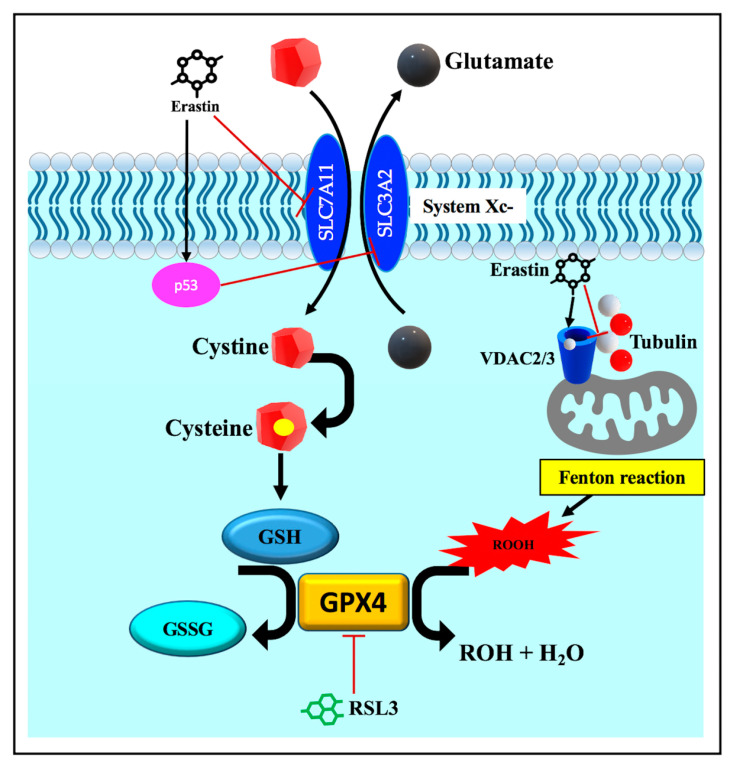
Schematic description of the endogenous redox homeostasis system, the Xc-GSH-GPX4 axis. The Xc-GSH-GPX4 axis is the major anti-ferroptosis defense mechanism. System Xc- imports cystine in exchange for glutamate. Cystine is reduced to cysteine, which is involved in the synthesis of GSH antioxidant. GPX4 enzyme reduces the products of the Fenton reaction (lipid peroxides) to non-toxic lipid alcohols at the expense of reduced GSH. Erastin interacts with system Xc- and VDAC2/3 mediating ferroptosis via increased ROS production. It also inhibits VDAC–tubulin interaction, attenuating aerobic glycolysis and increasing ROS generation. RSL3 mediates ferroptosis by direct inhibition of the GPX4 enzyme. The tumor suppressor gene p53 inhibits SLC7A11 transcription, interfering with GPX4-mediated antioxidant defense. RSL3 is a direct inhibitor for GPX4. Abbreviations: GPX4, glutathione peroxidase 4; GSH, glutathione; RSL3, RAS-selective lethal 3; ROH, alcohol; ROOH, peroxides; VDACs, voltage-dependent anion channels.

In agreement with the contribution of lipid peroxidation to ferroptosis, the impaired cellular capacity to eliminate lipid peroxides via the endogenous antioxidant defense system, including the Xc-glutathione (GSH)-glutathione peroxidase 4 (GPX4) axis system, is a characteristic feature of ferroptotic cell death [57]. The Xc-GSH-GPX4 system antagonizes ferroptosis by the elimination of ROS and lipid peroxides, which are involved in cellular demise. System Xc- is a plasma membrane heterodimer antiporter composed of two subunits, the light chain solute carrier family 7 member 11 (SLC7A11) and the heavy chain solute carrier family 3 member 2 (SLC3A2). These subunits drive the interchange of cystine, which presents extracellularly with glutamate, which itself presents intracellularly. The imported cystine is then reduced to cysteine, which is mandatory for the biosynthesis of the major intracellular antioxidant GSH [57]. The availability of GSH is required for the proper operation of the GPX4 enzyme. GPX4 is the sole glutathione peroxidase involved in the reduction in lipid peroxides to non-toxic lipid alcohols at the expense of reduced GSH (Figure 4) [57].

The role of these antioxidant pathways was initially identified after the application of ferroptosis inducers, such as erastin [5] and the RAS-selective lethal 3 (RSL3) molecules [21]. The erastin- or p53-mediated suppression of system Xc- induced the ferroptosis mode of cell death through the depletion of the major regulator of the redox condition of the cellular GSH (Figure 4) [58,59]. In contrast, ferroptosis prevention has been linked to the overexpression of SLC7A11 [5]. Moreover, downstream pharmacological (e.g., RSL3, Figure 4) and genetic disruption of GPX4 triggers ferroptosis, while resistance to ferroptosis is conferred by the overexpression of GPX4 [21,60,61]. Therefore, insufficiencies in Xc-, GSH, and GPX4, has been associated with the accumulation of lipid peroxides that react with intracellular labile iron, generating cytotoxic lipid radicals and mediating peroxidative damage to the biological membrane [62,63].

**Figure 5:**
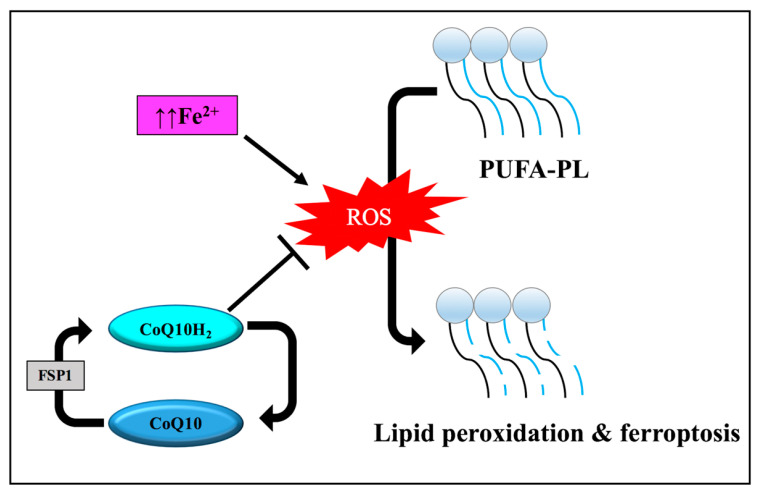
The role of suppressor protein 1 (FSP1) and coenzyme Q10 (CoQ10) in ferroptosis. FSP1 reduces CoQ10 to the antioxidant ubiquinol (CoQ10H_2_), preventing the generation of lipid peroxides and inhibiting ferroptosis. Abbreviations: PL, phospholipid; PUFAs, polyunsaturated fatty acids; ROS, reactive oxygen species.

Recent studies have identified an alternative pathway, other than GPX4, that is linked to lipid peroxide elimination and ferroptosis inhibition. The pathway involves both ferroptosis suppressor protein 1 (FSP1) and CoQ10, which is also known as ubiquinone. The FSP1 reduces CoQ10 to the antioxidant ubiquinol, preventing the generation of lipid peroxides and inhibiting ferroptosis (Figure 5) [64]. Therefore, the impaired intracellular antioxidant defense systems involved in scavenging lipid peroxides are one of the classical features of the ferroptotic form of cell demise. Considering the iron-dependent oxidative degradation of lipids, the execution of ferroptosis can be attenuated by the application of various iron chelators and antioxidants [46]. 

#### 3.3.4. Other Regulators of Ferroptosis

Ferroptotic cell death can be regulated by a number of additional factors other than those described in the previous sections. These factors generally affect the iron balance and alter lipid metabolism and peroxidation. Voltage-dependent anion channels (VDACs) have been found to play a role in controlling ferroptotic cell demise [65]. VDACs are major mitochondrial transmembrane channels that regulate the passage of metabolites and respiratory substrates between cytosol and mitochondria. Three isoforms of VDAC (VDAC1-3) have been identified; however, VDAC2/3 are considered positive regulators of ferroptosis [65]. It has been revealed that in addition to system Xc-, VDAC2/3 are the other direct drug-binding targets for erastin (Figure 4) [14]. Erastin–VDAC interaction mediates mitochondrial damage and the release of mitochondrial ROS that initiate iron-dependent ferroptotic cell death. Furthermore, the inactivation of VDAC2/3 has been associated with the suppression of erastin-mediated ferroptosis. Erastin–VDAC interaction can also block the interaction between VDAC and tubulin (Figure 4), resulting in the attenuation of aerobic glycolysis [14,66]. These observations support the concept that mitochondrial malfunction actively contributes to the initiation of ferroptotic cell death and suggest that cytoskeleton and energy metabolism are other important regulators of ferroptosis.

The tumor-suppressor gene *p53* has been characterized as a positive modulator of ferroptosis [58]. It inhibits SLC7A11 transcription (Figure 4), thereby reducing the amount of imported cystine and interfering with GPX4-mediated antioxidant defense. As a result, the missing GPX4 activity has been associated with excessive ROS generation and lipid-peroxidation-induced ferroptosis [58]. Recent studies have revealed that *p53* could also trigger ferroptosis via ALOX12 in the GPX4-independent ferroptosis pathway [24].

Finally, more research has emerged in recent years, demonstrating the involvement of HSF1 and HSPs in ferroptosis pathophysiology. These factors participate in the onset, development, and modulation of ferroptosis in distinct ways. The role of HSF1 and HSPs is discussed in more detail in the following section. 

Table 1 includes a brief history of molecular modulators of ferroptosis with examples of their pharmacological modulators.

## 4. The Role of Heat Shock Factor 1 (HSF1) and Heat Shock Proteins (HSPs) in Ferroptosis 

### 4.1. The Function of HSF1 in Ferroptosis

**Figure 6:**
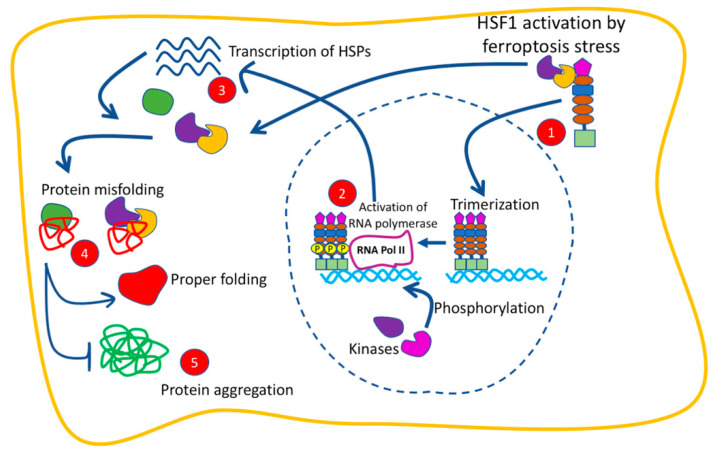
How HSF1 and HSPs act as anti-ferroptotic factors, preventing ferroptotic induced protein aggregation. When HSF1 is activated by ferroptosis stress, it trimerizes (step 1), which activates (after being phosphorylated by different kinases) RNA polymerase II (step 2), which then activates transcription of various HSPs (step 3). These HSPs then bind to misfolded proteins (step 4) and prevent them from aggregating (step 5).

Previous studies have identified that HSF1 controls several kinds of cellar death, including ferroptosis via multiple signal transduction pathways and the up-regulation of target genes (Table 2, Figure 6). Liu et al. revealed that co-treatment with erastin and another anticancer compound called celastrol induced ferroptotic cell death that was mediated by an increased ROS generation, mitochondrial fission, and mitophagy [82]. In conjunction with erastin and celastrol cotreatment, the transcriptional activation of HSF1 and its downstream effectors, HSPs, were observed. The study showed that HSF1-silencing was associated with improved cellular sensitivity to ferroptosis, whereas HSF1 overexpression attenuated ferroptotic cell death [82]. Other recent studies have shown that HSF1 could have been implicated in the regulation of ferroptosis via its interaction with other transcription factors involved in ferroptosis, such as p53. However, the precise mechanisms of such involvement have not been elucidated completely [79]. More recently, a study revealed that lipid peroxidation and ferroptosis in cardiac cells, which had been induced by palmitic acid (PA), were associated with the reduced expressions of HSF1 and GPX4 [83]. The reduced expressions of HSF1 and GPX4 observed in the study were improved by ferroptosis antagonists. The study also showed that HSF1 overexpression in cardiomyocytes interfered with PA-induced lipid peroxidation and ferroptosis by restoring GPX4 levels and modulating the expression of the genes responsible for iron metabolism. Consistently, treating HSF1-deficient mice with PA was associated with reduced GPX4 levels and more severe cardiomyocyte ferroptosis, as compared to the PA-treated wild-type mice. These observations suggested that HSF1 protects against ferroptosis via the maintenance of iron homeostasis and GPX4 activation [83]. 

### 4.2. The Function of HSPs in Ferroptosis

Molecular chaperones of the HSP70 family are highly conserved, ubiquitously expressed ATP-dependent proteins involved in several cellular processes linked to proteostasis, such as the folding of synthesized proteins, the refolding of misfolded polypeptides, the improvement of protein solubility, the inhibition of misfolded protein accumulation, the transport and translocation of protein-sequestering and degrading proteins, the regulation of their target proteins function, and the interaction with other HSPs members to maintain protein homeostasis [93]. HSP70 family members are highly related structurally and functionally and are identified in various cellular compartments, such as the cytosol, endoplasmic reticulum, and mitochondria. There are constitutive expression patterns for some HSP70 family members, such as HSP73 (also known as HSC70), and inducible expression patterns, which are dependent on certain triggering stimuli for other HSP70 family members, such as HSP72 [93]. The HSP40 is a DNAJ family protein that performs as a co-chaperone for HSP70s, regulating their ATPase function. Therefore, the HSP70 and HSP40 chaperone functions are somewhat interdependent. The HSP70 interacts with the N-terminal J domain of HSP40, stimulating the ATP hydrolysis that regulates the HSP70 function that maintains protein homeostasis [10]. 

The role of the HSP70 family proteins in the regulation of ferroptosis has been recently established (Table 2). The HSP70 protein 5 (HSPA5, also named binding immunoglobulin protein (BIP) or glucose-regulating protein 78 (GRP78)) has been recognized as a negative regulator of ferroptosis in several studies. Zhu et al. reported that HSPA5 prevented ferroptosis in pancreatic cancer. Zhu et al. demonstrated that the HSPA5 chaperone function protected GPX4 from degradation and the further accumulation of lipid peroxides in models of erastin-induced ferroptosis [75]. Similarly, dihydroartemisinin (DHA)-mediated ferroptosis in glioma cells was accompanied by the induction of the endoplasmic reticulum stress-signaling pathway, activating transcription factor 4 (ATF4) and HSPA5. The increased activity of HSPA5 had a protective effect against ferroptosis, as it potentiated the GPX4 expression and activity that prevented iron-dependent oxidative damage [84]. In support of the protective role of HSPA5 against ferroptosis, it has been shown that the suppression of the ATF4–HSPA5 axis in erastin-mediated ferroptosis in cancer cells enhanced the lipid peroxidation and ferroptotic cell death [75].

Little is known about the involvement of HSP40 in ferroptosis (Table 2). In contrary to the protective role of the HSP70 family against ferroptosis, a recent study reported that a DNAJ/HSP40 homolog, subfamily B, member 6 (DNAJB6, also known as the mammalian relative of DNAJ (Mrj)) had a pro-ferroptotic effect in esophageal squamous cell carcinoma. The study showed that the overexpression of DNAJB6 was associated with a decline in GSH levels, the downregulation of GPX4, and a consequent increase in lipid peroxidation [80].

The molecular chaperone HSP90 is a highly conserved protein expressed by eukaryotic cells [9]. HSP90 is an abundant protein that is expressed by multiple cellular compartments and represents 1–2% of the total cellular protein mass in cells that are not under stress. During stressful events, HSP90 cellular expression can increase up to 4–6% of its total cellular protein mass [94]. The protein structure of HSP90 is composed of 3 regions; the N-terminal domain that interacts with ATP; the central domain; and the C-terminal region that interacts with other client proteins and co-chaperones. HSP90 exists mainly in homodimers and the conformational changes in HSP90 dimers, and its interaction with target proteins is controlled by ATP catabolism and co-chaperoned binding [9,94]. HSP90 interacts with other chaperones, co-chaperones, and a large number of client proteins, such as tau, synuclein, kinases, and E3 ubiquitin ligases [9,94]. It is employed in the maturation and activation of newly synthesized proteins: the folding of proteins; the prevention of protein misfolding and the aggregation of proteins; and the degradation of peptides. This crucial chaperone function for HSP90 is important for different biological functions, such as cellular growth and differentiation, gene expression, the tracking of cellular components, and the maintenance of proteostasis [9,94]. Therefore, it is not surprising that this multifunctional molecular chaperone has been engaged in several pathological conditions, such as cancer and neurodegenerative disorders [9].

Recent studies have illustrated that HSP90, in association with other molecular chaperones and client proteins, has been employed in ferroptosis through the interference with the antioxidant function mediated by the GPX4 enzyme. Wu et al. reported that HSP90 functioned alongside another molecular chaperone HSPA8 (also known HSC70, a member of HSP70 proteins) to stimulate chaperone-mediated autophagy (CMA) that enhanced erastin-stimulated ferroptotic neuronal death. HSPA8 binds to GPX4, which is then recruited to the lysosome via the lysosome-associated membrane protein type 2a (LAMP2A). During this process, the HSP90 chaperone function is crucial for the stabilization of LAMP2A and the induction of the CMA-dependent lysosomal degradation of GPX4 and the further accumulation of lipid peroxides [78]. This HSP90-dependedent ferroptotic cellular death, mediated by erastin, is inhibited by HSP90 genetic-silencing and pharmacological inhibition [78]. Similarly, in models of acute kidney injury, the molecular chaperones HSP90 and HSC70 were involved in the CMA-mediated degradation of GPX4 [85]. To conclude, HSP90 has a significant role in controlling the function and the stability of numerous proteins implicated in ferroptosis (Table 2). This makes HSP90 an attractive potential therapeutic target in different pathological conditions.

HSPB1, also known as HSP27, is a member of the sHSPs family that is expressed in response to heat shock and other stressful stimuli in an HSF1-dependent manner [2]. HSPB1 is involved in various physiological functions as a molecular chaperone that interacts with several targeted cellular proteins to maintain proteostasis in an ATP-independent manner. The oligomeric structure and function of HSPB1 are regulated by post-translational modification, mainly phosphorylation [2]. HSPB1 interacts with protein components of the cytoskeleton, regulating their function, which includes cellular differentiation, cellular migration, and nutrient uptake [95]. 

The role of HSPB1 in ferroptosis has been identified after the discovery of its role in iron metabolism (Table 2). In particular, HSPB1 regulates the cytoskeleton-mediated iron uptake through its interaction with TFR1. It was found that the HSPB1-mediated stabilization of the cytoskeleton was associated with reduced cellular iron uptake through TFR1 [86]. Later studies revealed that erastin-induced ferroptosis in cancer cells was associated with an elevated HSPB1 expression in an HSF1-dependent manner [74]. Both heat shock and HSPB1 overexpression can suppress the execution of erastin-mediated ferroptosis, while HSF1- and HSPB1-knockdown drive the execution of erastin-induced ferroptosis [74]. Moreover, HSPB1 phosphorylation by the protein kinase C (PKC) inhibits ferroptosis by reducing the generation of iron-dependent lipid peroxides [74]. However, it has been elucidated that a compromised HSPB1 interaction with the cytoskeleton through impaired HSPB1 phosphorylation or defective polymerization of the cytoskeleton was associated with enhanced iron uptake and subsequent lipid peroxidation and ferroptotic cell death [74]. In conclusion, HSPB1 is considered a negative regulator of ferroptosis through its interaction with the cytoskeleton, reducing the cellular uptake of iron. Therefore, understanding the precise role of HSPB1 in ferroptosis could aid in developing anticancer therapies targeting either HSPB1 phosphorylation or cytoskeleton polymerization to modulate the iron uptake through the HSPB1–cytoskeleton interaction. 

HSPB5 is one of the well-studied sHSPs, after it was initially discovered as a major crystalline in the vertebrate lens. It is ubiquitously expressed and is involved in various cellular functions, such as the stabilization of the cytoskeleton, proteostasis control, anti-apoptotic activity, and neuroprotection [96]. Since HSPB5 plays several roles under physiological and stressed circumstances, it was not surprising that HSPB5 was found to be involved in various pathological conditions, such as cataracts, myopathies, cardiomyopathies, neurodegenerative disorders, and cancers [96]. However, little is known about the involvement of this sHSP in ferroptosis (Table 2), and a recent study revealed that chronic depression induction in a mice model was associated with the activation of ferroptotic neuronal death that involved the reduced expression of HSPB5 [81]; however, the exact role of the HSPB5 protein in ferroptosis is not yet fully understood and requires additional research.

HO-1, also called heat shock protein 32 (Hsp 32), is induced in response to several stressful events, such as heat shock, ROS, inflammation, heavy metals, and heme. The induction of HO-1 is controlled by a variety of transcription factors, including nuclear respiratory factor 2 (Nrf2), HSFs, nuclear factor–κB (NF-κB), nuclear factor–erythroid 2 (NF-E2), and activator protein–1 (AP-1), among others. HO-1 functions as an anti-oxidant and anti-inflammatory enzyme that catabolizes heme into biliverdin, carbon monoxide, and free iron [97,98]. 

The role of HO-1 in cellular death has been previously identified. Poss and Tonegawa indicated that the inactivation of murine HMOX1 was associated with renal and hepatic oxidative stress-mediated tissue damage [99]. More recent reports have described the link between HO-1 and ferroptosis (Table 2), in which HO-1 was shown to have dual effects in ferroptotic cellular death (Table 2). HO-1 was identified as a pro-ferroptotic factor in doxorubicin (DOX)-mediated ferroptosis in cardiomyopathy [87]; in EF24 (which is a curcumin analogue that functions as anti-tumor compound)-mediated ferroptosis in osteogenic sarcoma cells [88]; in sodium-iodate-induced ferroptosis in retinal degenerative diseases [89]; and in diabetic atherosclerosis [90]. In contrast, HO-1 displayed an anti-ferroptotic effect in erastin- and RSL3-mediated ferroptosis in renal epithelial cells [91], in models of acute kidney injury [100], and in models of neurodegenerative disorders [92]. Collectively, the regulatory function of HO-1 in ferroptosis is context-dependent; the pro-oxidative role of HO-1 may produce redox-active iron that is required for lipid peroxidation or it may provide a protective outcome through the activation of anti-oxidation mechanisms [101]. As a result, HO-1 can be identified as a prognostic factor in various pathological conditions linked to ferroptosis.

## 5. Conclusions and Perspectives

Iron is an important element in the body that is involved in several metabolic functions, such as oxygen transport and the catalysis of redox reactions. Because there is no iron disposal system, the body’s iron level is regulated by controlling the amount of iron absorbed and stored. The dysregulation of the iron balance in favor of iron accumulation reduces the capacity of cells to combat oxidative stress damage, culminating in membrane damage and leading to cellular death by ferroptosis. Generally, ferroptosis can be executed by the disruption of the Xc-GSH-GPX4 axis. There is growing evidence that the HSP molecular chaperones have a regulatory-role in iron-dependent ferroptotic cell death. In this review, we summarized the regulatory role that is played by several HSPs in ferroptosis (Table 2, Figure 6). Overall, HSPs have a dual role in ferroptotic cell demise, as some promote ferroptosis-resistance and others improve ferroptosis-sensitivity. These findings reflected an advancement in our comprehension of the different HSPs that cause ferroptosis; however, several questions remain: Why do different types HSPs behave inconsistently in the regulation of ferroptosis, that is, some have anti-ferroptotic roles while others have pro-ferroptotic roles? What are the factors that enhance the HSP-mediated CMA-dependent lysosomal degradation of GPX4? What is the role of iron chelators and antioxidants in HSPs-mediated ferroptosis? Since HSPs are involved in several cellular functions, what are the adverse effects of targeting pro-apoptotic HSPs to prevent the execution of ferroptosis, and how would this affect the general body proteostasis? Therefore, additional functional studies to elucidate HSPs’ mechanisms that contribute to ferroptosis are required to identify therapeutic targets in disorders related to ferroptosis. 

## Figures and Tables

**Table 1:** A brief history of molecular modulators of ferroptosis with examples of their pharmacological modulators.

Year of Identification	Ferroptosis Modulator	Function	Reference	Example on Pharmacological Modulator
2007	VDAC2/3	Mitochondrial transmembrane channels	[67]	Erastin & Piperazine [5,21]
	Mutated RAS	Oncogene	[67]	Erastin [5]
2008	TF	Cellular iron uptake	[68]	Siramesine [33] Lapatinib [33] Neratinib [69]
2012 (Identification of ferroptosis)	SLC7A11	Drives cystine glutamate exchange through System Xc-	[5]	Erastin & Piperazine [37] Sulfasalazine [5] Sorafenib & Glutamate [59]
2014	GPX4	Glutathione peroxidase involved in reduction in lipid peroxides	[21]	RSL3 [42] Altretamine [60] Withaferin A [70] DPI7 & Cisplatin [71]
2015	P53	Transcription factor	[58]	LncRNA P53RRA [72]
	ALOX12	Lipoxygenase	[24]	Baicalein [73]
	HSPB1	Molecular chaperone	[74]	Erastin [74]
	FTH1	Intracellular iron storage protein	[31]	-
	LPCAT	Lipogenesis	[42]	RSL3 & DPI7 [42]
2016	ACSL4	Lipogenesis	[22]	RSL3 & DPI7 [42]
2017	HSPA5	Molecular chaperone	[75]	-
	FPN	Iron transporter	[33]	-
2018	ALOXs	Lipoxygenases	[76]	Zileuton [77]
2019	HSP90	Molecular chaperone	[78]	2-amino-5-chloro-N,3-dimethylbenzamide (CDDO) [78]
	HSF1	Heat shock transcription factor	[79]	Erastin [74]
2020	DNAJB6	Molecular chaperone	[80]	-
	FSP1 & CoQ10	Antioxidant system	[64]	Altretamine & FIN56 [60]
2021	HSPB5	Molecular chaperone	[81]	-

**Table 2:** The role of heat shock factor 1 (HSF1) and heat shock proteins (HSPs) in ferroptosis.

Protein	Effect	Mechanism of Action	Reference
HSF1	Anti-ferroptotic	Erastin- and celastro-mediated ferroptosis is associated with increased ROS production, mitochondrial fission, autophagy, mitophagy, and transcriptional activation of HSF1. HSF1 reduces cellular sensitivity to ferroptosis induced by erastin and celastrol.	[82]
		HSF1 interacts with p53 transcription factor, supporting its transcriptional regulatory function in the cell cycle.	[79]
		Palmitic acid-induced ferroptosis is accompanied by reduced expression of HSF1 and GPX4. HSF1 protects against ferroptosis via maintenance of iron homeostasis and activation of GPX4 in palmitic acid-induced ferroptosis.	[83]
HSP70	Anti-ferroptotic	HSP70 protects GPX4 from degradation and potentiates its expression. This happens alongside the increased expression of HSPA5 (also known as BIP or GRP78).	[75,84]
HSP40	Pro-ferroptotic	HSP40 induction in esophageal squamous cell carcinoma is associated with decline in GSH levels and downregulation of GPX4.	[80]
HSP90	Pro-ferroptotic	HSP90, alongside HSPA8 (also known as HSC70), stimulates CMA-dependent lysosomal degradation of GPX4.	[78,85]
HSPB1 (also known as HSP27 and HSP25)	Anti-ferroptotic	HSPB1 mediates stabilization of the cytoskeleton and reduces cellular iron uptake through TFR1.	[86]
		Hsp27 phosphorylation by PKC inhibits ferroptosis by affecting iron metabolism. It decreases iron-dependent production of lipid ROS.	[74]
HSPB5 (also known as CRYAB)	Probably anti-ferroptotic	Chronic depression is linked to ferroptotic neuronal death and reduced expression of HSPB5. The mechanism of HSPB5 involvement in ferroptotic neuronal death is still unknown.	[81]
HO-1 (also known as Hsp 32)	Pro-ferroptotic	Doxorubicin-mediated ferroptosis in cardiomyocytes induces HO-1, which increases free iron production causing further lipid peroxidation of mitochondrial membrane.	[87]
		EF24-mediated ferroptosis in osteogenic sarcoma cells induces HMOX1 and increases malondialdehyde and ROS generation.	[88]
		Sodium-iodate-induced ferroptosis in retinal pigmented epithelium upregulates HO-1 with consequent free iron buildup and lipid peroxidation.	[89]
		Diabetic atherosclerosis is accompanied by HMOX1 induction that causes free iron accumulation, and further ROS production, and lipid peroxidation.	[90]
	Anti-ferroptotic	HO-1 inhibits erastin- or RSL3-induced ferroptosis in renal epithelial cells. The potential mechanism for HO-1 anti-ferroptotic effect in this model is that HO-1 upregulation provides antioxidant and cytoprotective effects in response to stressful stimuli.	[91]
		In neuronal cells, activation of HO-1 shows anti-ferroptotic function due to its antioxidant and anti-inflammatory function.	[92]

Abbreviations: BIP, binding immunoglobulin protein; CMA, chaperone-mediated autophagy; CRYAB, alpha-crystallin B; GPX4, glutathione peroxidase 4; GRP78, glucose-regulating protein 78; HO-1, HO-1/HMOX1, heme oxygenase -1; HSF1, heat shock factor 1; HSP27, heat shock protein 27; HSP40, heat shock protein 40; HSP70, heat shock protein 70; HSP90; heat shock protein 90; HSPA5, HSP70 protein 5; HSPA8, HSP70 protein 8; HSPB1, heat shock protein beta 1; HSPB5, heat shock protein beta 5; PKC, protein kinase C; ROS, reactive oxygen species; RSL3; RAS-selective lethal 3; TFR1, transferrin receptor 1.

## Data Availability

Not applicable.
